# Electrical Impedance Dermography: Background, Current State, and Emerging Clinical Opportunities

**DOI:** 10.1155/2024/2085098

**Published:** 2024-08-16

**Authors:** Elise K. Brunsgaard, Benjamin Sanchez, Douglas Grossman

**Affiliations:** ^1^ Huntsman Cancer Institute University of Utah Health, Salt Lake City, UT, USA; ^2^ Department of Electrical and Computer Engineering University of Utah, Salt Lake City, UT, USA; ^3^ Department of Dermatology University of Utah Health, Salt Lake City, UT, USA; ^4^ Department of Oncological Sciences University of Utah Health, Salt Lake City, UT, USA

## Abstract

Electrical impedance dermography (EID), based on electrical impedance spectroscopy, is a specific technique for the evaluation of skin disorders that relies upon the application and measurement of painless, alternating electrical current. EID assesses pathological changes to the normal composition and architecture of the skin that influence the flow of electrical current, including changes associated with inflammation, keratinocyte and melanocyte carcinogenesis, and scarring. Assessing the electrical properties of the skin across a range of frequencies and in multiple directions of current flow can provide diagnostic information to aid in the identification of pathologic skin conditions. EID holds the promise of serving as a diagnostic biomarker and potential to be used in skin cancer detection and staging. EID may also be useful as a biomarker in monitoring effectiveness of treatment in individual patients and in therapeutic research. This review highlights ongoing efforts to improve mechanistic understanding of skin electrical changes, study of EID in a variety of clinical contexts, and further refine the technology to find greater clinical use in dermatology and dermatologic research.

## 1. Introduction

While skin cancer screening has been improved by the addition of noninvasive techniques, methods such as dermoscopy and confocal microscopy require subjective interpretation and significant training for effective use [1–3]. In contrast, electrical impedance techniques, based on applying an alternating electrical current to a tissue and measuring the resultant voltages, offer an objective and quantitative alternative to evaluate suspicious lesions [4]. The term electrical impedance dermography (EID) refers specifically to the application of electrical impedance techniques to evaluate skin disorders [5]. EID provides a quick and painless method to assess electrical changes in the normal architecture of the skin across a range of frequencies of electrical current. Previous studies have shown its potential as an adjunct diagnostic tool when applied to pre-cancerous and cancerous skin lesions [5–11]. In addition, EID may be useful for assessing skin barrier function and can provide valuable information on treatment efficacy in conditions such as atopic dermatitis [12–14]. There has been limited review of EID in dermatology, and previous work focused specifically on its use in skin cancer detection [15]. This article aims to discuss the basic electrical properties of the skin, review the progression of device development for measuring electrical impedance, and highlight a range of clinical and research applications of EID.

## 2. Electrical Impedance Dermography

### 2.1. Basic Electrical Impedance Principles

**Figure 1:**
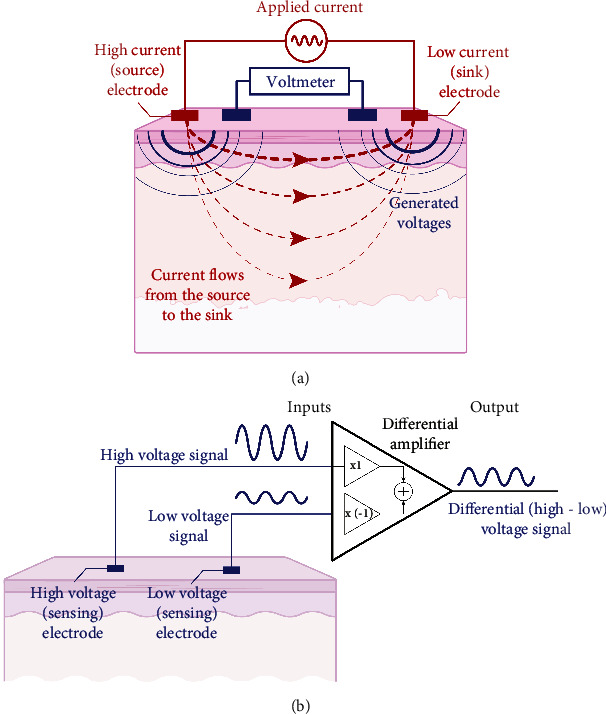
Simplified illustration representing the principles of an EID measurement. (a) Electrical current is applied between the source and sink electrodes, whereas the resultant generated voltages are picked by the sensing electrodes. (b) Schematic of a differential amplifier used to amplify the voltage difference sensed by the voltage electrodes. The common voltage present at the electrodes is cancelled by the amplifier.

Electrical impedance methods are based on the application of alternating electrical current and the measurement of generated electrical voltage (Figure 1(a)) [16]. As the electrical current flows through tissues, the amplitude of the voltage measured will be altered due to the extracellular and intracellular ionic media. Due to cells' ability to create a potential difference by holding electrical charges on both sides of the membrane, the timing of the voltage waveform will experience a time delay. This time delay will result in a phase shift in the measured voltage signal with respect to the applied electrical current. The two voltage waveforms detected by the sensing electrodes are fed into a differential amplifier (Figure 1(b)). The differential amplifier has two inputs, designated as noninverting and inverting inputs. The noninverting input multiplies the high-voltage signal by a positive gain, whereas the inverting input inverts the low-voltage signal with respect to its polarity (by multiplying the signal by a negative gain). The signals detected by the high- and low-voltage sensing electrodes are thus first amplified by the same amount and then summated electronically by the differential amplifier. As a result, any voltage signals that are common to both electrodes are effectively subtracted while those that are different are amplified and subsequently used to calculate the impedance. At various frequencies of electrical current, the voltage-current signals' peak amplitude ratio and their time-lag relationship are used to determine the apparent electrical impedance of the tissue using Ohm's law [4], i.e., impedance equals voltage divided by current. The measured impedance is determined by two quantities, its resistance *R* and reactance *X* components or, equivalently, its magnitude *M* and phase *P*, via the trigonometric relationships $M=\sqrt{R^{2}+X^{2}}$ and *P*=tan^−1^ *X*/*R*, or *R*=*M* cos *P* and *X*=*M* sin *P*.

### 2.2. Representation of EID Data

**Figure 2:**
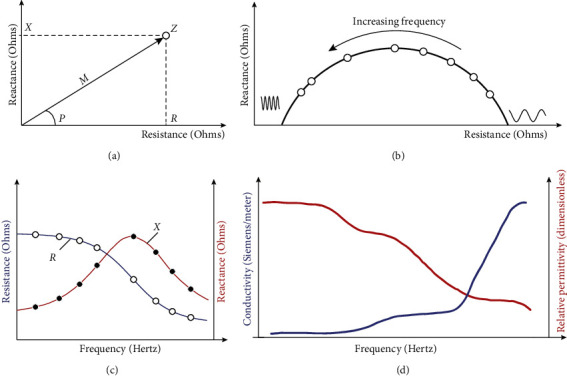
Schematic illustrating the representation of EID data. (a) Diagram showing the relationship between EID data, resistance *R*, and reactance *X*, and their equivalent counterparts, magnitude *M* and phase *P*. (b) Representation of *X* versus *R* at various frequencies of electrical current. (c) Equivalent representation of *R* and *X* against the frequency. (d) Representation of the conductivity and relative permittivity properties of skin against the frequency.

When EID reactance and resistance data are plotted against each other (Figure 2(a)), the impedance *Z* is represented as a dot where the *x* and *y* coordinates are *R* and *X*, respectively. The length of the line that goes from the coordinate origin to the impedance is the impedance magnitude *M*, and the angle *P* is the inclination of this line. At various frequencies of electrical current, *X* represented against *R* describes an arc of a circumference (Figure 2(b)). Equivalently, *R* and *X* can be represented explicitly against the frequency (Figure 2(c)). Sweeping across frequencies, the resistance *R* will exhibit monotonically decreasing dependence with increasing frequency, whereas the reactance *X* will show a bell-like curve.

### 2.3. Relationship between EID Data and the Underlying Skin Electrical Properties

From EID, it is also possible to solve for *R* and *X* data and infer the underlying electrical properties of the skin, namely, the conductivity and the relative permittivity properties, including their directional dependence, a concept known as electrical anisotropy [5]. When plotted against frequency, conductivity has a monotonically increasing dependence on frequency, while relative permittivity is the opposite (Figure 2(d)).

Skin conductivity is a measure of how skin resists or conducts alternating electrical current, whereas skin relative permittivity is a measure of its capacity to store electrical charge [17]. Measured skin *R* and *X* values reflect a complex combination of skin underlying electrical conductivity and relative permittivity properties. Structural changes that occur as a result of disease pathology alter the ionic and cellular components of the skin, affecting its conductivity and relative permittivity properties and impacting the EID measurements.

### 2.4. Instrumentation and Measurement Principles of EID

EID measurements are made over a small area of interest, and the outcomes are objective, quantitative values that provide an electrical characterization of the skin. If skin conductivity and relative permittivity values are reported, then these outcomes are standardized and can be compared between studies. Although it is also possible to obtain an electrical impedance image of the skin, this technology is not readily available and its application has been minimally explored [18].

**Figure 3:**
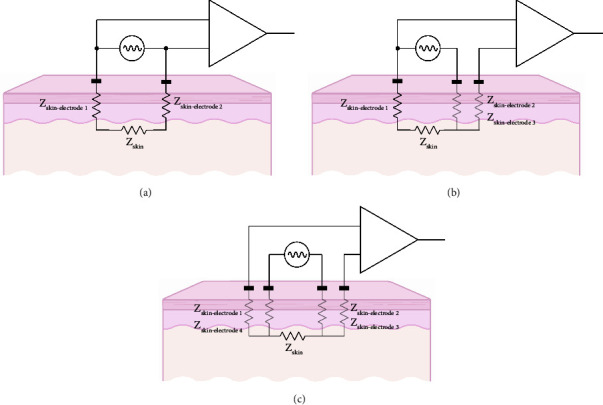
Basic EID recording circuits, consisting of a current source and differential amplifier. (a) Two-electrode setup. (b) Three-electrode setup. (c) Four-electrode setup. Note only the impedances in black color contribute to the overall EID data measured.

A minimum of two electrodes are needed to perform EID measurements (Figure 3(a)). The first electrode (current source) applies electrical current to the skin and the second electrode (current sink) closes the electrical circuit, allowing the current to find its way back to the device. At the same time, the two electrodes are used to measure the voltage signal, and their difference is amplified by the differential amplifier. In a two-electrode configuration, the measured impedance is the sum of the skin impedance plus the polarization impedance resulting from the contact between the two electrodes with the skin.

Measurement complexity may be increased by the addition of a third electrode (Figure 3(b)) [19]. In a three-electrode configuration, the measured impedance is the sum of the skin impedance plus the polarization impedance resulting from the contact between the current source electrode and the skin. By using a third independent voltage electrode, the voltage measured is not affected by experimental electrode artifacts that may occur at the sink current electrode (e.g., temperature drifts or poor electrode contact with the skin).

Importantly, two- and three-electrode setups are limited in that measurement of skin impedance also contains the skin-electrode polarization impedances resulting from the contact between the current electrodes and skin. Skin-electrode polarization impedance is a poorly controllable experimental factor where alterations in skin-electrode contact area, skin humidity, or temperature can give large impedance variations between measurements and corrupt readings, particularly at low frequencies where the skin-electrode polarization impedance is larger [20]. Skin-electrode impedance polarization artifacts can be effectively mitigated by adding a fourth electrode (Figure 3(c)), in which independent pairs of electrodes are used to apply current and measure voltage which results in measuring only the electrical impedance of the skin. [21].

### 2.5. Electrodes

Conventional, noninvasive wet electrodes use gels and often provide the best impedance recordings. However, skin irritation with wet electrodes can be significant, and their large size limits their spatial resolution [22]. Noninvasive dry electrodes typically cause less skin irritation and only require the skin to be moistened with saline to improve the electrical contact between the electrode and the skin [23]. Dry electrodes also have an advantage over wet electrodes in that they can be constructed to measure small skin lesions [23]. Minimally invasive electrode arrays penetrate the stratum corneum which reduces the electrode-skin contact impedance and improves the accuracy of measurements compared to noninvasive electrode arrays. However, the design of these electrodes is more complex and expensive to manufacture [24].

## 3. Historical Perspective

Historically, electrical impedance techniques have been widely used for nondestructive characterization applications in engineering and biosciences, but more recently they have been studied for potential clinical applications. The use of EID to differentiate normal and abnormal skin conditions has gained traction over the past 30 years (Table 1). Early impedance devices were used in research investigating the skin barrier and skin irritation in the cosmetic industry [25, 26]. Subsequent research evaluated electrical impedance differences in basal cell carcinoma (BCC) [27, 29]. Further development of impedance techniques which penetrate the stratum corneum allowed for more accurate identification of melanoma [10, 30]. EID also shows promise in objectively evaluating skin barrier function and has been used in studies to assess the efficacy of treatments for conditions such as atopic dermatitis [12, 14, 38].

## 4. Clinical Applications

### 4.1. Limitations of Existing Noninvasive Imaging Technologies

While accurate diagnosis of skin cancer requires biopsy and histologic confirmation, there are several noninvasive modalities available to facilitate visual examination of deeper structural components of the skin. Dermoscopy is primarily used for pigmented lesions to assist in the identification of melanoma, but it can also be used to identify features associated with BCC [39] and squamous cell carcinoma (SCC) [40]. However, even in expert hands, dermoscopy has a sensitivity and specificity for BCC of approximately 80% with limited ability to distinguish between BCC subtypes [1]. Similarly, even with dermoscopy, SCC in situ can exhibit overlapping features with invasive SCC and inflamed seborrheic keratosis (SK) [41].

Confocal microscopy allows for direct visualization of histologic structures beneath the skin surface and can facilitate the identification of both BCC [42] and SCC [3]. However, this technology has significant limitations, including visualization only to the depth of the superficial dermis, high cost, and extensive training required for image interpretation [2]. There is interest in applying machine learning for the interpretation of clinical and dermoscopic or confocal images, but existing systems trained on two-dimensional photographs have not performed well in real-world clinical settings [43]. Therefore, there is a need for more objective, easy-to-use devices to assist in the assessment of concerning lesions.

### 4.2. Electrical Properties and Management of Skin Cancer

Based on the biophysical electrical properties of skin, the histologic differences between nonmelanoma skin cancer (NMSC), melanoma, actinic keratosis (AK), and SK should impact the flow of electrical current through the lesions and consequently affect skin impedance values measured by EID in four-electrode setups. For example, alterations in composition and tissue structure associated with NMSC and SK (e.g., epidermal hyperplasia, tumor cells in epidermis and dermis, stroma in BCC, keratin pearls in SCC, and UV-induced elastosis and infiltration of inflammatory cells) are predicted to change the ionic content of the skin and will affect its electrical conductivity. However, there is limited research characterizing the distinct electrical properties of the different subtypes of NMSC or melanoma.

#### 4.2.1. Nonmelanoma Skin Cancer

NMSC, specifically BCC and SCC, is the most prevalent type of cancer with an estimated five million cases diagnosed in the United States annually [44]. BCC arises from the basal layer of the epidermis while SCC arises from the suprabasal squamous layers. When diagnosed at an early stage, NMSC can usually be treated by destruction or topical therapy, but larger or deeply invasive tumors require surgical excision that may be associated with substantial morbidity [45, 46].

There are multiple subtypes of BCC that can be difficult to distinguish clinically, and biopsy is required for optimal histologic analysis and therapeutic decision-making [46]. Histologically, the superficial form of BCC is confined to the epidermis and the nodular form consists of round collections of tumor cells occupying the upper part of the dermis. The more invasive BCC subtypes, micronodular and infiltrative (or morpheaform), consist of smaller aggregates of tumor cells or angulated or stranded tumor cells, respectively, infiltrating the deeper dermis. The invasive forms of BCC cannot reliably be distinguished clinically from nodular or superficial subtypes of BCC.

Similar to BCC, superficial and invasive forms of SCC require a biopsy to differentiate. SCC in situ is differentiated from invasive SCC by depth of invasion histologically. SCC in situ can often be treated nonsurgically (although SCC in situ that involves hair follicles may be associated with higher rates of recurrence) while invasive SCC usually requires surgical excision [45]. Having a real-time ability to differentiate superficial versus invasive subtypes of BCC and SCC would be invaluable for informing treatment decisions.

#### 4.2.2. Melanoma

Melanoma is the deadliest form of skin cancer, and melanoma-specific survival improves significantly when detected at earlier stages [47]. Melanoma develops from uncontrolled melanocyte proliferation in the epidermis, and cells may infiltrate into the upper layers of the epidermis and dermis. Melanoma does not impact the stratum corneum to the same extent as NMSC. However, minimally invasive EID techniques have been successful in identifying melanoma with high sensitivity [7, 10, 11]. The four main subtypes of melanoma are superficial spreading, nodular, acral lentiginous, and lentigo maligna. Each has distinct histologic features that would be expected to impact electrical impedance; however, there are no published studies on EID in different melanoma subtypes to date.

### 4.3. Benign Skin Cancer Mimics

Another potential clinical application of EID is differentiating skin cancers from benign mimics. SK is a benign neoplasm characterized by epidermal hyperplasia [48]. These lesions occasionally become inflamed and might be tender or exhibit peripheral erythema. These histologic changes can make it difficult to distinguish an inflamed SK from SCC in situ. Multiple studies of EID have reported high false-positive rates for SK due to the impedance measurements being similar to NMSC and SK being incorrectly identified as malignant [6, 7].

### 4.4. Efficacy of EID Devices in Skin Cancer Identification

Multiple studies have found significant, reproducible electrical impedance differences between skin cancer and normal skin or nevi using both commercially and internally developed EID devices. The most used device, Nevisense (SciBase AB, Stockholm, Sweden), is a system initially designed to measure pigmented lesions to distinguish melanoma from nevi, but it has been effectively applied to detect NMSC [8, 9, 36]. Nevisense was preceded by SCMI, SciBase I, II, and III. The different EID devices discussed are outlined in Table 2.

The ability to differentiate skin cancer from normal skin or nevi was established by multiple preclinical and early clinical studies [27, 29]. A study of 35 BCC utilizing SciBase I examined differences between impedance measurements of nodular (*n* = 15) and superficial (*n* = 20) subtypes of BCC [31]. However, they found no significant differences in the impedance measurements between the two BCC subtypes [31]. A study of noninvasive versus micro-invasive EID techniques using SciBase II found that noninvasive probes were the most effective in separating BCC (*n* = 28) from nevi with 86% specificity at 96% sensitivity, whereas micro-invasive probes were more effective when distinguishing melanoma (*n* = 13) from nevi with 80% specificity at 92% sensitivity [30]. A study of 511 lesions using SciBase II found that skin cancer could reliably be differentiated from nevi with a reported 75% specificity at 100% sensitivity for melanoma (*n* = 16) and 87% specificity at 100% sensitivity for NMSC (*n* = 94) [10]. In a subsequent multicenter study using SciBase II, an automated classification algorithm to distinguish between melanoma and benign lesions showed an observed sensitivity of 95% (59/62) and overall specificity of 49% (72/148) [11].

Two large, multicenter studies provided evidence for the Nevisense system as an adjunct tool for skin cancer screening [6, 7]. The primary aim of the first study was to develop a classification algorithm for differentiating melanoma from benign lesions, and two different algorithms were tested [6]. SciBase III was used to measure a total of 1,300 lesions. The observed sensitivities for the first and second algorithms for melanoma were 98.1% (101/103) and 99.4% (161/162), respectively [6]. The study also identified NMSC with 100% (25/25; 21 BCC, 4 SCC) sensitivity for the first algorithm and 98% (49/50; 39 BCC, 11 SCC) sensitivity for the second algorithm [6]. The overall specificities for the two algorithms were 23.6% and 24.5%, respectively [6]. The second study used Nevisense to measure 1943 pigmented lesions and reported an observed sensitivity for melanoma of 96.6% (256/265) and an overall specificity of 34.4% [7]. The study also identified 48 BCC and 7 SCC with a reported 100% observed sensitivity for NMSC overall [7].

The findings of these larger trials were replicated in subsequent studies focused on the use of Nevisense to identify NMSC. A study of 200 lesions reported a sensitivity of 94% for NMSC, missing three BCCs, and a specificity of 43% [8]. Similarly, a study of over 1700 suspicious lesions found an observed sensitivity of 100% for both BCC (82/82) and SCC (13/13) [9]. EID measurements were found to strongly correlate with clinical and dermoscopic grading of AK with an observed sensitivity of 98% (49/50) [36].

In addition to the SciBase devices, several other EID devices have been used to differentiate skin cancer from normal skin. A single probe with a matrix of electrodes (TransScan) showed that impedance changes were associated with melanoma tumor growth in mice and histopathologic findings in human melanoma lesions with 67% specificity and 92% sensitivity [28].

In addition, a pilot study of 17 BCCs using a four-electrode EID device (URSKIN) found significant, reproducible electrical impedance differences between BCC and normal skin [5]. More recently, a pilot study using URSKIN on 35 skin lesions was able to differentiate SCC in situ from inflamed SK and normal skin (*p* < 0.001) [37]. Unlike the Nevisense, URSKIN takes only 30 seconds for measurements, and data are transmitted directly from the device to a smartphone for data visualization and analysis.

### 4.5. Clinical Biopsy Decision-Making

The integration of EID into clinical practice could be beneficial for improving the clinical decision-making process for biopsy and treatment of suspicious lesions. Two survey-based studies evaluated the impact of EID measurements on biopsy decisions [49, 50]. In the first study, 267 dermatologists were shown images of 43 pigmented lesions (16 were melanomas) and asked whether biopsy was indicated based on clinical appearance alone. They were then provided with corresponding EID measurements and asked if the information changed their biopsy decisions. After incorporating the impedance scores into their clinical decision-making process, the number that needed biopsy improved from 6.3 to 5.3 (*p* < 0.001), sensitivity from 84% to 98% (*p* < 0.001), and specificity from 34% to 44% (*p* < 0.001) [49]. The second study of 164 dermatology trainees found that providing the impedance scores with the clinical images (45 pigmented lesions; 17 were melanomas) altered the biopsy decision in 24.3% of cases, and the addition of the score resulted in 402 fewer missed melanomas and an overall decrease of 376 biopsies of benign lesions (*p* < 0.001) [50]. The sensitivity for ruling out melanoma increased from 80.7% to 95.2% (*p* < 0.001) and specificity from 50.4% to 58.6% (*p* < 0.001) [50]. However, a significant limitation of both studies was the use of clinical images rather than in vivo examination and the omission of clinical information that would be collected in a typical visit.

Another study evaluated the impact of adding baseline EID measurements for suspicious melanocytic lesions to inform who would benefit most from short-term digital dermoscopy imaging follow-up [33]. Of the 160 lesions, 6 were determined to be melanoma, and of those monitored by imaging, one was later determined to be melanoma. The observed sensitivity and specificity were both 83%. Using the proposed cutoff scores, the need for dermoscopic imaging follow-up was decreased by 47% [33]. These studies suggest that when used for clinically suspicious lesions, EID has the potential to increase the effectiveness of skin cancer screening by improving detection of malignancy and decreasing unnecessary biopsies and intensive follow-up.

### 4.6. Epithelial Tissue Barrier

EID has been shown to be comparable to more established techniques such as measuring total epidermal water loss and offers an alternative method to assess skin barrier function. An EID study using Nevisense in atopic versus nonatopic skin found significant changes in impedance scores between atopic and healthy skin [12]. The study determined that impedance-based techniques were useful in detecting skin reactivity and evaluating lipid content in the stratum corneum in atopic skin [12]. Similarly, another study assessing lesional and unaffected skin in patients with atopic dermatitis found that increased EID scores correlated with healing and decreased severity and itch scores, demonstrating that EID measurements were reliable for evaluating skin barrier dysfunction [14]. A study evaluating the change in biophysical properties of atopic skin following the application of an emollient showed normalization of impedance indices following treatment [13]. The ability to objectively measure atopic dermatitis severity and monitor treatment response would be particularly useful in the development of new therapeutics.

EID has been used to assess skin hydration, mechanical damage, and response to UV irradiation. Studies evaluating skin hydration dynamics [35] and changes in impedance following mechanical damage to the skin barrier [32] provide a rationale for using EID in transdermal drug delivery research. In addition, EID measurements have been found to correlate with macroscopic tissue damage in UV-treated pig skin membranes. [34] These studies suggest that EID could be used to determine the sun protection factor value for sunscreens and assess their efficacy.

## 5. Conclusions and Perspectives

Given the promising findings of studies using electrical impedance-based techniques, EID has demonstrated potential as an adjunct skin cancer screening tool, a reliable method to assess treatment response in atopic dermatitis, a clinical research device to study transdermal drug delivery, and a measurement tool to assist in sunscreen formulation. Larger datasets, further development of measurement techniques, and new machine learning analytics are warranted to enable accurate differentiation of skin cancer mimics from malignant lesions and discrimination between NMSC subtypes.

## Figures and Tables

**Table 1:** Summary of studies using EID.

Published [reference]	Type	Clinical application	Device
1983 [19]	Preclinical	Nonspecific use assessing skin impedance	Two electrodes with portable meter
1992 [25]	Clinical (*n* = 11)	Skin irritation	Single probe
1996 [26]	Clinical (*n* = 28)	Skin irritation	Surface-characterizing impedance monitor (SCIM) prototype
1998 [27]	Clinical (*n* = 12)	Nodular BCC	Surface-characterizing impedance monitor (SCIM)
2003 [28]	Preclinical (mice, *n* = 40), clinical (*n* = 178)	Melanoma, BCC	TransScan
2003 [29]	Clinical (*n* = 258 lesions)	BCC	SciBase I
2004 [10]	Clinical (*n* = 611 lesions)	Nevi, AK, BCC, SCC, melanoma	SciBase II
2004 [12]	Clinical (atopic, *n* = 26; nonatopic, *n* = 22)	Atopic dermatitis	SciBase I
2005 [30]	Clinical (*n* = 140)	Nevi, BCC, melanoma	SciBase II
2005 [31]	Clinical (*n* = 35)	Nodular and superficial BCC	SciBase I
2006 [13]	Clinical (atopic dermatitis, *n* = 24; controls, *n* = 22)	Atopic dermatitis	SciBase I
2011 [11]	Multicenter, clinical (*n* = 285 lesions)	Melanoma	SciBase II
2013 [6]	Multicenter, clinical (*n* = 1,300 lesions)	BCC, SCC, melanoma	SciBase III
2013 [32]	Preclinical (human cadaver skin)	Skin barrier– mechanical damage	Gamry potentiostat (model PCI4/300, Warminster, PA)
2014 [7]	Multicenter, clinical (*n* = 2,416 lesions)	BCC, SCC, melanoma	Nevisense
2017 [33]	Clinical (*n* = 160 lesions)	Melanoma	Nevisense
2019 [34]	Preclinical (pig skin)	UVB irradiation	Four-electrode setup mounted in a Franz cell
2020 [35]	Preclinical (pig skin), clinical (*n* = 12)	Skin hydration	Nevisense
2020 [8]	Clinical (*n* = 200 lesions)	AK, BCC, SCC	Nevisense
2021 [9]	Clinical (*n* = 1,712 lesions)	AK, BCC, SCC	Nevisense
2021 [14]	Clinical (atopic dermatitis, *n* = 36; control, *n* = 28)	Atopic dermatitis	Nevisense
2021 [36]	Clinical (*n* = 50)	AK	Nevisense
2022 [5]	Clinical (*n* = 17)	BCC	URSKIN (University of Utah, Salt Lake City, UT)
2023 [37]	Clinical (*n* = 35)	SCC, inflamed SK	URSKIN (University of Utah, Salt Lake City, UT)

AK: actinic keratoses; BCC: basal cell carcinoma; SCC: squamous cell carcinoma; SK: seborrheic keratosis.

**Table 2:** Advantages and limitations of EID devices.

Device	Location	Description	Advantages	Limitations
Surface-characterizing impedance monitor (SCIM) [27]	Kinna, Sweden	Single probe with four concentric electrodes	(i) Measures magnitude and phase (ii) 31 frequencies at 5 depths	(i) Depth limited to 2 mm (ii) Must be connected to a laptop to operate

TransScan [28]	Migdal Haemek, Israel	Single probe with 8 × 8 matrix of gold-coated electrodes that penetrate the stratum corneum	(i) Measures conductivity and capacitance (ii) Minimally invasive electrodes that allow for the early detection of melanoma	(i) Must be connected to a laptop to operate

SciBase I [29]	Huddinge, Sweden	Noninvasive depth selective probe with four concentric electrodes	(i) Measures magnitude and phase angle (ii) Can differentiate NMSC from nevi	(i) High impedance variance within groups (ii) Electrical interference distorting measurements

SciBase II [10, 30]	Huddinge, Sweden	Depth selective probe with four concentric electrodes attached to a ceramic plate or minimally invasive probe	(i) Improved signal-to-noise ratio compared to SciBase I with minimally invasive version (ii) Minimally invasive form can reliably differentiate melanoma from nevi	(i) Interference from stratum corneum with the noninvasive probe when assessing melanoma

SciBase III [6]	Huddinge, Sweden	Spring-loaded probe with a five-bar electrode	(i) Implemented classification algorithm to improve identification of melanoma versus nevi (ii) 35 frequencies at 4 depths	(i) Lesions must be >5 mm

Nevisense [7]	Stockholm, Sweden	Spring-loaded probe with minimally invasive five-bar electrode	(i) Multiple large validation studies (ii) Noninvasive	(i) Expensive (ii) Long time to measure (>15 minutes) (iii) Large instrument size (iv) Lesions must be >5 mm

URSKIN (University of Utah) [5]	Salt Lake City, UT	Noninvasive 16-electrode array	(i) Less than 5 minutes per measurement (ii) Small instrument size (iii) Measures depth up to 7 mm	(i) Requires further validation (ii) Lesions must be >5 mm

## Data Availability

Raw data is available to qualified investigators upon request.
